# Supplementary material for: Phylogenetic analysis of Mycobacterium bovis reveals animal and zoonotic tuberculosis spread between Morocco and European countries
Source: PLoS Negl Trop Dis. 2025 Feb 18;19(2):e0011982. doi: 10.1371/journal.pntd.0011982 (PMC11892842; doi:10.1371/journal.pntd.0011982)
Supplement: Attachment — Submitted filename: PNTD_Letter to the reviewer.docx [file pntd.0011982.s006.docx]

PNTD-D-24-00221

Phylogenetic analysis of *Mycobacterium bovis* reveals evidence of animal and zoonotic tuberculosis spread between Morocco and European countries.

We would like to thank the editor and reviewers for your valuable comments on our manuscript. We have included answers to every question as well as a copy of the edited text in the manuscript.

1) *Line 160-162, Sample collection, it is not well explained why the duration of sample collections vary among the sites, how would you explain the possible risk of selection bias in sample collection?*

We agree with the reviewer that this is a point that needs to be strengthen. The duration of sample collections varied among sites, and we have edited the methods section to clarify both the duration of sampling, and the number of samples collected from each site. We also clarified which already existing samples were added to our collection and which ones had metadata available.

[Lines 167-174]: “The collection period for Rabat was during March and April 2015 and for El Jadida was from June 2014 to July 2015. Samples collected from Oujda do not have information about the collection date. The length difference in sampling periods resulted in different numbers of isolates per location, where four were collected from Rabat, thirty-eight from El Jadida, and nine from Oujda (however, as mentioned above, no collection dates were available for this location). Four other *M. bovis* collected from cattle were added to the collection, but these had no metadata available.”

We agree that the difference in sampling duration during Morocco samples collection could have affected the number of collected samples per site and create risk of sampling bias. This would be a problem if we were performing a comparison across sites in Morocco. However, in our study, we analyzed the samples from the different collection sites together and compared them with samples from other countries, therefore, the possible sampling bias across sampling locations does not change the integrity of our results that show *M. bovis* phylogenetic links between cattle in Morocco and cattle and humans in European countries. It is important to notice, though, that the genetic diversity found in our samples is not representative of the genetic diversity and clonal complexes existing in Morocco. Our study found one new spoligotype in Morocco, but there might be more, and more systematic sampling of *M. bovis* is needed to determine the overall *M. bovis* genetic diversity in the country. We have provided more information about data collection in the materials and methods section (lines 174-188), and discussed this limitation in the discussion section (lines 429-449).

[Lines 174-188]: “The difference of sampling period between Rabat and El Jadida was due to the lack of a country-wide surveillance program for bTB in Morocco and due to logistics related to each study site such as the availability of veterinaries to assist in the data collection and/or authorized transportation to the site. However, these differences in sampling size are not important factor for our current study since we are not performing a comparison across study sites in Morocco, but a comparison between *M. bovis* samples from Morocco and samples from other countries.”

[Lines 429-449]: “One of the limitations of the study is that the duration of collection of Moroccan *M. bovis* samples included in the analysis was different across the collection sites, which could have influenced the overall genetic diversity found in Morocco, and therefore, not be representative of the full genetic diversity of *M. bovis* in the country and its relatedness to neighboring countries. However, in our study, we analyzed the samples from the different collection sites together and compared them with samples from other countries, therefore, the possible sampling bias across sampling locations does not change the integrity of our results that show *M. bovis* phylogenetic links between cattle in Morocco and cattle and humans in European countries. It is important to notice that the genetic diversity found in our samples is not representative of the genetic diversity and clonal complexes existing in Morocco. Our study found one new spoligotype in Morocco, but there might be more. As such, it is needed more systematic sampling over a longer period of time to be able to capture the overall genetic diversity and clonal complexes in Morocco. Additionally, the human samples included in the analysis lacked attached metadata, making it difficult to draw any definitive epidemiological conclusions about *M. bovis* cattle to human transmission.”

2) *Figure 3 and figure 4 quality are very poor and cannot be read.*

We have uploaded new high-resolution versions of both figures 3 and 4. Both figures have been inspected and processed by PACE (https://pacev2.apexcovantage.com/; recommended by the publisher) to generate figures with the requested publisher specification recommendations.

In addition, we have

- replaced the term ‘transmission’ with ‘spread’ in the title
- corrected the number of animals for each sex category and breed type in the Materials and Methods section
- added a Supporting Information file with the captions of Supporting Files 1 and 2
- edited Supporting File 2 to add Bioproject Accession Number and Biosample Acession Numbers of each sequence
