# Supplementary material for: Phylogenetic analysis of Mycobacterium bovis reveals animal and zoonotic tuberculosis spread between Morocco and European countries
Source: PLoS Negl Trop Dis. 2025 Feb 18;19(2):e0011982. doi: 10.1371/journal.pntd.0011982 (PMC11892842; doi:10.1371/journal.pntd.0011982)
Supplement: Attachment — Submitted filename: Review.docx [file pntd.0011982.s003.docx]

**Journal: PLOS, Neglected Tropical Disease**

**Article No: PNTD-D-24-00221**

Thank you for the manuscript.

**Major comments:**

1) Line 36: “Bovine tuberculosis (bTB), mainly caused by Mycobacterium bovis (M. bovis)” the sentence gives the impression that there are also some other causes for bTB also.
2) Line 38: You have mentioned “in addition to the unknown human health burden caused by zoonotic transmission” I would suggest to go through literature again. I have provided an example: <https://pubmed.ncbi.nlm.nih.gov/27697390/>
3) Line 54: The term “North Africa” also includes Morocco. How would you justify the sentence “no M. bovis sequences from North Africa in the present database were classified”????
4) The data that which is provided in the supplements is poorly analyzed.
5) In sample collection it is not clearly explained that, why would sample collection duration vary between regions?
6) Line 71: “…….contact with animal carcasses and infected organs at slaughter houses” based on the reference that you provided it has reported: “2101 cases of pulmonary TB from the Cheshire sanatorium represented the period 1934–1943. Of these, just 48 cases (2.28%) were bTB and only 10 were strongly suggestive of airborne transmission from cattle”. This is not aligned with what you have written.
7) line 72: The comparison of bTB cost between Morocco and Ethiopia is not quite convincing, how would you justify this comparison?
8) The discussion part is not well organized and does not present a clear message. There are many controversies in the discussion part which are not clearly explained.
9) What are the strengths and limitations of this study? It has been known for decades that *mycobacterium* *bovis* can be transmitted from animals such as bovine to humans, what knowledge or implementation gap you have addressed in this manuscript?
10) There is no conclusion part to summarize the findings and gaps for future research.

**Minor comments:**

1) line 36: “and food safety and security.” No need for two “ands”
2) Introduction, line 63: “decreased animal health and economic distress in many countries of the world”, the term “many countries” is not specific, I would suggest to be more specific on it.
3) Sample collection is not clearly explained in terms of choosing the sample. Was the examiner looking for all organs and inspecting the organs separately or only the animals’ lungs?
4) Table-1, “Areas of origin” it is not clearly understandable if the mentioned areas are the where animals were imported? Or Slaughtered?
5) For mycobacterial culture and genome sequencing part: No references are provided. This makes it unclear whether you followed WHO/CDC guidelines or any other SOPs.
6) EU-1, EU-2, EU-3 and AF1 are poorly explained and reader may not understand clearly about it.
7) Table-1: you have provided data about 52 samples from different parts of Morocco but in discussion you have mentioned 56 isolated.
8) In the sample collection you have mentioned “Rabat, El Jadida (western Morocco) and Oujda (Eastern Morocco)” but in discussion you have related them all to the north of Morocco. The reason for choosing the north of Morocco is also poorly explained in the text.
9) line 340-341: you have mentioned that the closest match of Moroccan cattle to human isolates were from Germany, UK and Italy but in the discussion part, you have stated that: the results showed that isolates from Morocco are genetically related to isolates from Spain, France. How would you explain this?
10) line 80: based on the reference you provided it has mentioned “North America” not the USA.
11) line 86: it is important to mention at what number or proportion it has been isolated from Eurasian wild boar.
12) line 93 is in opposition with line 80, see comment 5.
13) line 96: “2012 in Morocco, bTB herd prevalence was 56%” based on your reference the percentages are different with what you have reported. *“20.4% (95% CI 18%-23%) and 57.7% (95% CI 48%-66%), respectively, were observed in this study*.”
14) line 109-117: The reference for this paragraph is missing however, above these paragraph 5 references are given for a single sentence. (line 108-109)
15) line 130: space is needed after “[19]”.
16) Line 183: “Cattle with gross visible lesions suggesting bTB were sampled”. Based on what was explained earlier the examiner has visited the slaughterhouse and taking sample from the slaughtered animals’ organs. This is not in aligned with your sampling.
17) line 254: In total 780 genomes were analyzed but in line 273 it says 784. Is there any thing missing?
