# Supplementary material for: Phylogenetic analysis of Mycobacterium bovis reveals animal and zoonotic tuberculosis spread between Morocco and European countries
Source: PLoS Negl Trop Dis. 2025 Feb 18;19(2):e0011982. doi: 10.1371/journal.pntd.0011982 (PMC11892842; doi:10.1371/journal.pntd.0011982)
Supplement: Attachment — Submitted filename: reviewers letter_August28.docx [file pntd.0011982.s004.docx]

PNTD-D-24-00221

Phylogenetic analysis of *Mycobacterium bovis* reveals evidence of animal and zoonotic tuberculosis transmission between Morocco and European countries.

We would like to thank the reviewers for your valuable comments. We included explanations and edits to answer every comment and question. Following are general edits and below are specific answers to all the reviewers’ comments (in red)

- The introduction has been shortened, and the objectives of the study were clearly defined and re-aligned with the content of the paper.
- All the different sections of the manuscript have been restructured to follow reviewer 2 comments. These sections are now separated into 1) describing the genetic diversity of *M. bovis* from cattle from Morocco, 2) *M. bovis* from cattle in Morocco compared to *M. bovis* from cattle and wildlife in other countries, and 3) *M. bovis* from cattle in Morocco compared to *M. bovis* from humans in other countries.
- Table 1 was deleted, and information summarized in a paragraph.
- The Map was edited to include an outline that shows the location of the Sahara Desert
- Figure 2 was deleted from the manuscript.
- Limitations and strengths of the study, as well as suggestions for future work were added to conclusion.

Reviewer's Responses to Questions

Reviewer #1:

1) objectives are clear but the article has deviated from main objectives.

*The manuscript has been edited to focus on the objectives of the study and to follow the same structure in the results and discussion sections.*

2) Population is not clearly defined.

*For the first part of our analyses, we have defined the population of focus of our study in Morocco, which is the cattle population that is slaughtered in 3 slaughterhouses (Rabat, Casablanca, and Oujda) and have suspected lesions of tuberculosis infection. From these carcasses, we extracted infected tissues, cultured Mycobacterium bovis from them, and performed whole-genome sequencing of 55 Mycobacterium bovis isolates. For the second part of the analyses, we defined our group of isolates as any Mycobacterium bovis isolate that was publicly available from NCBI that had metadata links with Morocco either through evidence of cattle trade or human migration.*

Reviewer #2:

1. Objectives "to determine the genetic relatedness and epidemiological links" comes late in the Introduction and should be moved to the opening paragraphs. this focuses the reader better.  The introduction is too long and could be restructured to explain: i) the impact of M bovis in Morocco for animal health (with limited economic data from nearby Ethiopia) ii) the potential for M bovis to be spread to humans and wildlife iii) initial molecular analysis using older methods iv) the role of WGS in clarifying genetic relationships

*We have edited the introduction based on your suggestion (see lines 85-153).*

2. Study design is adequate, but vulnerable with the highly variable comparison data from isolates outside Morocco.  The design might be reformatted to present Moroccan cattle isolates compared to a: other cattle, b: human isolates, c: wildlife?

*We have edited the results and discussion sections to follow the suggested structure. We first covered the relationship between M. bovis isolates between cattle from Morocco and cattle and wildlife from other countries, then compared the relationship between M. bovis isolates between cattle from Morocco and humans from other countries. We opted to not include a separate section for comparisons between M. bovis isolates between Moroccan cattle and wildlife from other countries since no genetic links were found between them, so we presented the results related to cattle and wildlife from other countries together.*

--------------------

Reviewer #1: The results are not clearly presented and the reader may find it challenging to get to the main point.

*For improved clarity, we have structured the Results section based on Reviewer 2 suggestions: Comparison of M. bovis isolates between cattle in Morocco and cattle and wildlife from other countries, then comparison of M. bovis isolates between cattle in Morocco and humans from other countries. We have also moved the clonal complex results to the top of the Results section to first highlight the M. bovis genetic diversity based on spoligotyping and clonal complexes, followed by the phylogenetic analysis of the different isolates to study the M. bovis genetic relationship between Morrocan cattle and animal and humans from countries that are spatially adjacent and/or have trade and/or migration histories with Morocco.*

The tables are ok but the dataset is not analyzed adequately.

*Following Reviewer 2’s suggestion, we have deleted Table 1, since the data provided were not directly related to the goals and analyses of the manuscript.*

Reviewer #2:

1. the results are challenging to present, since the Moroccan isolates and the reference isolates are so different.

*We have restructured the manuscript to present our results in a more organized and clear manner. In this new version, we first highlight the M. bovis genetic diversity based on spoligotyping and clonal complexes (turning Figure 5 into Figure 2) and we focused the phylogenetic analysis of the different M. bovis isolates to study the M. bovis genetic relationship between Moroccan cattle isolates and the isolates from animal and humans from countries that are spatially adjacent and/or have trade and/or migration histories with Morocco. This new structure puts Moroccan isolates as the center of the comparison and directly addresses the goals of the study. We have also redid Figure 1 to highlight the Sahara desert on the map to visually show the spatial separation between North Africa (Morocco and Algeria) and Sub-Saharan Africa, and Figure 5 (new Figure 2) to improve the clarity of the figure and highlight one of the consequences of the physical separation between North Africa and Sub-Saharan Africa: the clonal complexes found in the newly sequenced Moroccan isolates are all from “European” or “Unkonwn” clonal complexes, instead of being from “African” clonal complexes, as we could initially expect.*

2. Table 1 does not help the manuscript, and could be summarized in a paragraph.

*We have deleted Table 1 and summarized its contents in a paragraph.*

Figure 1 map is OK, but adding the location of Sahara Desert would be helpful.

*Thank you for the suggestion, we have added an outline for the Sahara Desert in the map.*

12 of 52 animals were imported from where? could importation have any

relevance to their genetic analysis?

*This sentence refers to the importation of Holstein breed cattle, which Morocco imports from several European countries: France, Germany and Spain as mentioned in the Introduction. We did find genetic links between Mycobacterium bovis isolates from cattle in Morocco and cattle in these countries, which suggests that is due to trade between these countries. However, and unfortunately there is no specific cattle traceability information available for national and international trade.*

Figure 2 is unreadable. Only the outer circle has numbers; zooming in only shows blurred characters.  This Figure should be removed.

*This figure has been deleted as suggested.*

Figure 3 contains important data, but is almost too much for the reader to comprehend.  

*We thank the reviewer for the concern, we agreed that this figure has a lot of information, however, because our goal is to relate the metadata with the phylogeny, we decided to keep all the data together to avoid redundancy in extra figures. We redesigned the figure to improve clarity for the reader by putting together all the figure legends and increasing their boxes and text size. We have also increased the text of group number to highlight the different group numbers in the phylogeny.*

Figure 4 contains the most important data the authors are presenting.  This should be made into 7 or 8 individual Figures, so that the Discussion can address each Panel properly and separately.  Group 6 has no comments, and could be excluded?

*Figure 4 was previously separated into 8 different figures, but the captions and the discussion section became very redundant, therefore we decided to merge them into one figure with different panels, which are explained in detail in the figure caption. To improve the clarity for the reader, we edited the caption of the figure focusing on the relationship of Mycobacterium bovis isolates between Moroccan cattle and cattle, wildlife, and humans in other countries. We added information for Panel 6 to the caption illustrating that no genetic relationship involving Morocco was found.*

Figure 5 is tangential data, and does not contribute to the article.

*We have edited Figure 5 (now Figure 2), which presents data supporting the clonal complex distribution between North Africa and Sub-Saharan Africa. We find this result significant since it shows that the genetic diversity of Mycobacterium bovis isolates is different from the Sub-saharan African since no African clonal complexes were detected in the Moroccan data. This supports that the Saharan desert is playing a large role in the circulation of M. bovis across Africa and that North Africa isolates are actually more similar to the European ones.*

The WGS analysis is easier to comprehend than the lineage and clonal complex data.

*We have restructured the manuscript to improve clarity and added the clonal complex results at the beginning of the results section to introduce the fact that M. bovis clonal complexes in Morocco are more like the ones in Europe than to the ones in Sub-Sahran Africa. This result provides a good introduction and support of our hypothesis that M. bovis isolates from Morocco are more linked to European ones through animal trade and/or human migration than to Sub-Saharan Africa.*

--------------------

Reviewer #1: No conclusion is present. 

Limitations and strengths of the study are not reported. It is also not mentioned what knowledge or implementation gap exactly they have addressed.

*We have added limitations and strengths of the study to the Discussion section, please see line 392-396.*

Reviewer #2: The conclusions show the strength of WGS analysis in revealing connections among isolates from a wide variety of sources. Again the conclusions could be segregated into sections of a: cattle to cattle comparisons, b: cattle to human, and c: cattle to wildlife.

*We have restructured the paper and the Discussion focusing on the suggested subsections. The cattle-wildlife relationship does not have its own subsection throughout the manuscript because no links were found between M. bovis isolates extracted from Moroccan cattle and isolates from wildlife in other countries.*

This is an initial attempt at WGS analysis, and the comparison data set is so different making comparisons challenging.

*We agreed that that there are so many more analyses that can be done with this large dataset and we added that information in the future directions of this work (lines 397-402). The comparisons of isolates became much easier after following the reviewers’ suggestions and focusing on the relationships between cattle-cattle, cattle-wildlife and cattle-humans. We highlighted the Moroccan isolates and made the suggested comparison types throughout the text, which in our opinion, have improved the manuscript considerably.*

Public health relevance is paramount for this kind of manuscript, and needs considerably more emphasis of both the animal and human health implications.

*We agree with the reviewer and added a paragraph in the discussion section*

*on the public health evidence and the role of One Health in this type of work. (see lines 345-357), and we have mentioned the intent to use the One Health approach in future studies (Line 399)*

--------------------

<b>Editorial and Data Presentation Modifications?</b></br><br/>
Use  this section for editorial suggestions as well as relatively minor modifications of existing data that would enhance clarity. If the only modifications needed are minor and/or editorial, you may wish to recommend “Minor Revision” or “Accept”.

Reviewer #1: Minor comments:

1) line 36: “and food safety and security.” No need for two “ands”

Changed to food safety and food security.

2) Introduction, line 63: “decreased animal health and economic distress in many countries of the world”, the term “many countries” is not specific, I would suggest to be more specific on it.

We thank the reviewer for this suggestion. We changed the term “many countries” to “low- and middle-income countries”.

3) Sample collection is not clearly explained in terms of choosing the sample. Was the examiner looking for all organs and inspecting the organs separately or only the animals’ lungs?

The samples were collected following routine meat inspection at the slaughterhouse, where all the organs and the whole animal carcass were inspected. Samples were collected from lesions that were suggestive of *Mycobacterium bovis* infection. We have clarified this information in the manuscript in lines 164-165.

4) Table-1, “Areas of origin” it is not clearly understandable if the mentioned areas are the where animals were imported? Or Slaughtered?

We have removed Table 1 as suggested and only left the description of the animals’ demographic information in the text.

5) For mycobacterial culture and genome sequencing part: No references are provided. This makes it unclear whether you followed WHO/CDC guidelines or any other SOPs.

The SOP followed for mycobacterial culture is the one that has been developed in our lab and published in (<https://bmcvetres.biomedcentral.com/articles/10.1186/s12917-017-1165-6>) . For the whole-genome sequencing, we followed the USDA guidelines (<https://www.ncbi.nlm.nih.gov/pmc/articles/PMC8370811/>) and we used their publicly available bioinformatics pipeline vSNP (https://github.com/USDA-VS/vSNP) to process the outputted sequences.

6) EU-1, EU-2, EU-3 and AF1 are poorly explained and reader may not understand clearly about it.

*We added information about clonal complexes in the Introduction section and have restructured the Methods, Results and Discussion sections to clarify this topic. Furthermore, we moved Figure 5 into Figure 2 to highlight the importance of clonal complex classification and their geographical distribution.*

7) Table-1: you have provided data about 52 samples from different parts of Morocco but in discussion you have mentioned 56 isolated.

*The metadata associated to the isolates were not available for 4 of the 56 animals, and one isolate did not have high enough DNA quality to be sequenced, so the total of the sequenced isolates from Morocco was 55. We clarified this information in the manuscript in the following sections: lines 166-170.*

8) In the sample collection you have mentioned “Rabat, El Jadida (western Morocco) and Oujda (Eastern Morocco)” but in discussion you have related them all to the north of Morocco. The reason for choosing the north of Morocco is also poorly explained in the text.

*We have removed Western and Eastern Morocco from the text. The reason we mentioned the North of Morocco in the discussion is that the South of Morocco is part of the Sahara Desert, so we meant the general north of the country.*

9) line 340-341: you have mentioned that the closest match of Moroccan cattle to human isolates were from Germany, UK and Italy but in the discussion part, you have stated that: the results showed that isolates from Morocco are genetically related to isolates from Spain, France. How would you explain this?

*Thank you for calling our attention to this issue. The close genetic relationship between Morocco and the three countries Germany, UK and Italy refers to cattle M. bovis (from Morocco) to human M. bovis (from the 3 countries), while the relatedness of cattle M. bovis isolates from Morocco to isolates from Spain and France refers to cattle M. bovis exclusively. We have restructured the discussion to improve clarity (lines 297-385).*

10) line 80: based on the reference you provided it has mentioned “North America” not the USA.

*We have deleted this section from the manuscript, since the information was not aligned with the objectives of the study.*

11) line 86: it is important to mention at what number or proportion it has been isolated from Eurasian wild boar.

*This information has been added to the manuscript (*lines 332-333*).*

12) line 93 is in opposition with line 80, see comment 5.

*This section has been deleted from the manuscript since the information was not aligned with the objectives of the study*.

13)percentages are different with what you have reported. “20.4% (95% CI 18%-23%) and 57.7% (95% CI 48%-66%), respectively, were observed in this study.”

line 96: “2012 in Morocco, bTB herd prevalence was 56%” based on your reference the

*We thank the reviewer for alerting for these typos. The herd prevalence has been corrected, please see lines 110-111.*

14) line 109-117: The reference for this paragraph is missing however, above these paragraph 5 references are given for a single sentence. (line 108-109)

*We have edited the reference appropriately.*

15)  line 130: space is needed after “[19]”.

*Done.*

16)  Line 183: “Cattle with gross visible lesions suggesting bTB were sampled”. Based on what was explained earlier the examiner has visited the slaughterhouse and taking sample from the slaughtered animals’ organs. This is not in aligned with your sampling.

*This has been better explained in the Methods section. Indeed, the inspectors collected samples from cattle with gross visible lesions suggesting bTB, please see line* 164-165*.*

17) line 254: In total 780 genomes were analyzed but in line 273 it says 784. Is there any thing missing?

*This was a typo. The total is 780 genomes and we have corrected the number.*

Reviewer #1: Major comments:

1) Line 36: “Bovine tuberculosis (bTB), mainly caused by Mycobacterium bovis (M. bovis)” the sentence gives the impression that there are also some other causes for bTB also.

*Yes, that is correct. Mycobacterium bovis is the main cause of bovine tuberculosis, however, recent studies have shown that bovine tuberculosis cases in cattle have been caused by M. tuberculosis and M. orygis (*[*https://www.ncbi.nlm.nih.gov/pmc/articles/PMC9973683/*](https://www.ncbi.nlm.nih.gov/pmc/articles/PMC9973683/)*) , (*[*https://www.frontiersin.org/articles/10.3389/fvets.2021.691192/full*](https://www.frontiersin.org/articles/10.3389/fvets.2021.691192/full)*). We added this information to the manuscript for improved clarity.*

2) Line 38: You have mentioned “in addition to the unknown human health burden caused by zoonotic transmission” I would suggest to go through literature again. I have provided an example: https://pubmed.ncbi.nlm.nih.gov/27697390/.

*We have replaced the unknown burden with “underestimated”, as mentioned in the suggested paper.*

3) Line 54: The term “North Africa” also includes Morocco. How would you justify the sentence “no M. bovis sequences from North Africa in the present database were classified”????

*We meant to say that no M. bovis isolates from North Africa used in this study were classified as AF1 or AF2 clonal complexes. We edited the sentence to improve clarity on this topic (lines xx).*

*“The fact that no M. bovis sequences from North Africa in the present database were classified as AF1 or AF2 clonal complexes suggests that the Sahara might play a role in preventing M. bovis transmission between North Africa and Sub-Saharan Africa”.*

3) The data that which is provided in the supplements is poorly analyzed.

*The data provided in the supplements has been edited to have just a list of all the isolates analyzed in the present manuscript, which are the most relevant data for the analyses performed. We have deleted all the other data from the Supplementary Material since it was not needed.*

5)  In sample collection it is not clearly explained that, why would sample collection duration vary between regions?

The duration of the sample collection varied between regions because xxx

*We have added a sentence to explain the difference in sample collection duration between the regions, which was mostly due to the lack of a systematic bTB surveillance system in Morocco and to the logistics related to travel to the slaughterhouse and the availability of a professional veterinarian to collect the samples and send them to our laboratory.*

6) Line 71: “.......contact with animal carcasses and infected organs at slaughter houses” based on the reference that you provided it has reported: “2101 cases of pulmonary TB from the Cheshire sanatorium represented the period 1934–1943. Of these, just 48 cases (2.28%) were bTB and only 10 were strongly suggestive of airborne transmission from cattle”. This is not aligned with what you have written.

Thank you for bringing this to our attention. we have removed the sentence “contact with animal carcasses and infected organs at slaughterhouses” since it is an unlikely transmission route, even if it’s possible. We opted to delete it to avoid confusion.

7) line 72: The comparison of bTB cost between Morocco and Ethiopia is not quite convincing, how would you justify this comparison?

*Unfortunately, there is no estimation of the bTB cost in Morocco or in most low-income countries. The only similar setting for which this calculation has been done is Ethiopia, which has large similarities to Morocco (LIC, no systematic surveillance for bTB, bTB endemic). We have included this information in the introduction to provide an idea of the high economic losses due to bTB.*

8) The discussion part is not well organized and does not present a clear message. There are many controversies in the discussion part which are not clearly explained.

*We have restructured the discussion to follow the same general flow of the results sections as was suggested by the reviewer.*

9) What are the strengths and limitations of this study? It has been known for decades that mycobacterium bovis can be transmitted from animals such as bovine to humans, what knowledge or implementation gap you have addressed in this manuscript?

*We have added a paragraph to the Discussion section that summarizes the strengths and limitations of the study.*

10) There is no conclusion part to summarize the findings and gaps for future research.

*We have added a paragraph to the Discussion section that summarizes the findings and gaps for future research.*

Reviewer #2: The strength of this paper is the enormous amount of molecular data has been obtained for analysis.  But the execution is somewhat rambling and would benefit from reformatting the Introduction, Results and Discussion into subsections of cattle - cattle; cattle - human and cattle - wildlife datasets.

*We thank the reviewer for the recognition of the large dataset of our study. We have followed your suggestion and have reformatted the Introduction, Results and Discussion in the format that you proposed.*
