# Supplementary material for: Phylogenetic analysis of Mycobacterium bovis reveals animal and zoonotic tuberculosis spread between Morocco and European countries
Source: PLoS Negl Trop Dis. 2025 Feb 18;19(2):e0011982. doi: 10.1371/journal.pntd.0011982 (PMC11892842; doi:10.1371/journal.pntd.0011982)
Supplement: Attachment — Submitted filename: Review-2.docx [file pntd.0011982.s005.docx]

**Journal: PLOS, Neglected Tropical Disease**

**Article No: PNTD-D-24-00221**

Thank you for the manuscript. Overall, the manuscript has been adapted based on the comments. The structure of the discussion is now clear. There are only two minor comments which I would strongly recommend checking them and adapt.

Thanks for the hard work

**Minor comments:**

1) Line 160-162, Sample collection, it is not well explained why the duration of sample collections vary among the sites, how would you explain the possible risk of selection bias in sample collection?
2) Figure 3 and figure 4 quality are very poor and cannot be read.
